# Cancerous Tumor within the Antrum—Removal of Superior Maxillary Bone under the Influence of Chloroform

**Published:** 1851-04

**Authors:** 


### ARTICLE XII.

*Cancerous Tumor within the Antrum—Removal of the Superior Maxillary Bone under the Influence of Chloroform.*

M. VIGUERIE, senior surgeon of the Hotel Dieu of Toulon, recently performed the following operation:

The patient, a maiden lady, forty-five years old, had suffered for several months from great pain in the eye, which organ offered no appearance of any lesion whatever. After soothing means had been used some time, it became clear, from the protrusion of the eye, that it was pushed forward by an exostosis within the orbit: but it was some time afterwards discovered that there was much difficulty in the passage of the air through the corresponding nostril, and at last no doubt remained as to the cause of the mischief. A tumor of either a polypous or sarcomatous description was developing itself within the antrum, and having found resistance to its development towards the tuberosity of the maxilla, it was pushing the floor of the orbit upwards, the eye forwards and outwards, and extending into the nasal fossæ. M. Viguerie then perceived that nothing but the removal of the whole superior maxilla could offer any chance to the patient. This last resource, however, was, from some circumstances independent of the surgeon's control, delayed for some time, during which the affection made considerable progress. The nasal orifice was plugged by a sarcomatous mass, and another was projecting under the skin, between the nose and the inner canthus of the eye. It was resolved that the method of M. Gensoul, of Lyons, should be adopted, and the operation took place on the 30th of December, 1847. Chloroform was used, by the patient's request: about one drachm, on a sponge, sufficed to throw her, in thirty seconds, into a quiet sleep.

A rectangular flap was made on the cheek by three incisions, of which the first descended vertically from the inner extremity of the eyebrow to the upper lip, in the direction of the canine tooth; the second, being external, ran from the center of the malar bone, a little below the outer canthus of the eye, vertically to meet the third, which was horizontal, and united the two first a little below the ala nasi. The operator had first intended to save the mouth, but he found that in doing this, he would be very confined as to reaching the mesial line, and he was obliged to cut through the lip. The square flap was quickly dissected and raised to level with the orbital margin, where it was held by an assistant. The surgeon then introduced an eyed and curved stilette into the inferior and external angle of the orbit, and made it return towards himself through the spheno-maxillary fissure. The extremity of the stilette had been provided with a thread, by means of which the chain saw was pulled after it, and the malar bone was sawed at the point where it joins the maxillary. At this moment the patient woke, and became much agitated, evidently not knowing what was going on; she was at last soothed down again, the chloroform sponge was re-applied, and the operation proceeded, the patient remaining very quiet, without, however, falling into a torpid state. The nasal process of the maxillary bone being much softened, and involved in the cancerous growth, it was sufficient to detach it carefully from the surrounding parts; the palate process was separated from its healthy fellow by a section along the mesial line, performed with the straight chisel and mallet. The maxillary bone had still firm connections backwards, and it was impossible to attack the pterygoid process by the oblique section recommended by M. Gensoul; the floor of the orbit was so raised as not to allow the introduction of the chisel along it, for fear of wounding the eye. M. Viguerie then resolved to attempt the removal of the bone by moving it strongly from above downwards; and he succeeded after a little resistance. The sub-orbital nerve, and all the surrounding soft parts, were then divided, and the cancerous growth, which had invaded the internal parietes of the orbit, was very easily removed. The septum narium was then quite apparent; its mucous membrane looked red and healthy: sarcomatous masses, which had reached up to the frontal sinus, were removed by the index finger of the operator. As the upper part of the pterygoid process had been broken, in the efforts to remove the maxillary bone, the velum was hanging loosely on that side, and required to be pared off with the scissors; an artery which then bled freely was stopped by torsion; other portions of this cavernous wound were treated with the actual cautery, and cold water was afterwards syringed upon the parts. The patient, who had now full consciousness, easily rejected the blood and mucus which were falling into the pharynx. The flap was then brought down, and secured to the surrounding part by needles and twisted sutures; the lip was also united very carefully by the same process. The examination of the tumor confirmed the diagnosis: it was an encephaloid growth, which originated in the antrum, and had progressed as above mentioned.

The patient having been placed in bed, cold compresses were applied to the wound. No hemorrhage ensued, but sanguineous mucus continued for a few days to be discharged from the mouth. Slight traumatic fever supervened. On the eighth day the external wound was cicatrised; a little swelling persisted along the nose; all the needles were gradually removed as cicatrization proceeded. The eye was still prominent. The patient had stated that for a few hours after the operation, vision had been improved; but on the 20th of January, severe pain in the eye came on; profuse suppuration followed; the cornea broke on the 28th, and much matter, previously pent up in the anterior chamber, escaped. The eye was subsequently lost; but excepting this drawback, the condition of the case, at the time of the last report, was satisfactory—speech had become intelligible, the patient swallowed tolerably well, and very little deformity remained.

